# Nutritional Value and Volatile Compounds of Black Cherry (*Prunus serotina*) Seeds

**DOI:** 10.3390/molecules20023479

**Published:** 2015-02-17

**Authors:** Leticia García-Aguilar, Alejandra Rojas-Molina, César Ibarra-Alvarado, Juana I. Rojas-Molina, Pedro A. Vázquez-Landaverde, Francisco J. Luna-Vázquez, Miguel A. Zavala-Sánchez

**Affiliations:** 1 Doctorado en Ciencias Biológicas y de la Salud, Universidad Autónoma Metropolitana, Unidad Xochimilco, Mexico, D.F. 04960, Mexico; E-Mail: laetitia_spp@yahoo.com; 2 Laboratorio de Investigación Química y Farmacológica de Productos Naturales, Facultad de Química, Universidad Autónoma de Querétaro, Querétaro, Qro. 76010, Mexico; E-Mails: cibarra@uaq.mx (C.I.-A.); jirojasmolina@gmail.com (J.I.R.-M.); fjlunavz@yahoo.com.mx (F.J.L.-V.); 3 Centro de Investigación en Ciencia Aplicada y Tecnología Avanzada del Instituto Politécnico Nacional, Unidad Querétaro. Cerro Blanco No. 141. Col. Colinas del Cimatario, Querétaro, Qro. 76090, Mexico; E-Mail: pavazquez@ipn.mx; 4 Departamento de Sistemas Biológicos, Universidad Autónoma Metropolitana, Unidad Xochimilco, México, D.F. A.P. 23-181, Mexico; E-Mail: mzavala@correo.xoc.uam.mx

**Keywords:** *Prunus serotina* seeds, black cherry seeds, volatile compounds, proximal analysis, amino acid profile

## Abstract

*Prunus serotina* (black cherry), commonly known in Mexico as capulín, is used in Mexican traditional medicine for the treatment of cardiovascular, respiratory, and gastrointestinal diseases. Particularly, *P. serotina* seeds, consumed in Mexico as snacks, are used for treating cough. In the present study, nutritional and volatile analyses of black cherry seeds were carried out to determine their nutraceutical potential. Proximate analysis indicated that *P. serotina* raw and toasted seeds contain mostly fat, followed by protein, fiber, carbohydrates, and ash. The potassium content in black cherry raw and toasted seeds is high, and their protein digestibility-corrected amino acid scores suggest that they might represent a complementary source of proteins. Solid phase microextraction and gas chromatography/flame ionization detection/mass spectrometry analysis allowed identification of 59 and 99 volatile compounds in the raw and toasted seeds, respectively. The major volatile compounds identified in raw and toasted seeds were 2,3-butanediol and benzaldehyde, which contribute to the flavor and odor of the toasted seeds. Moreover, it has been previously demonstrated that benzaldehyde possesses a significant vasodilator effect, therefore, the presence of this compound along with oleic, linoleic, and α-eleostearic fatty acids indicate that black cherry seeds consumption might have beneficial effects on the cardiovascular system.

## 1. Introduction

*Prunus serotina Ehrh* (American black cherry), commonly called “capulín” in Mexico, is a native North American tree that belongs to the family *Rosaceae* [1]. It grows wildly or under cultivated conditions in Mexican highlands and in some regions of Guatemala, Colombia, and Venezuela [2]. In recent times, it has spread widely in some European countries, like Belgium, the Netherlands, and Germany [3]. *P. serotina* is a very fast-growing tree which has an oval silhouette, its low branches normally droop and touch the ground, the leaves are dark green and shiny; the fruit is a little juice drupe about 1 cm long with a stone which contains a seed [4].

In Mexico, *P. serotina* has been used since colonial times for nourishment and medicinal purposes. The fruit is commonly consumed fresh, as jam, liquors or syrups, and the seeds are consumed roasted and salted as snacks [2]. In Mexican traditional medicine, teas and syrups prepared from the leaves and fruits are highly appreciated for treating hypertension, stomach upsets, mouth infections, diarrhea, malaria, bronchitis, and cough [5,6], and the seeds are used in decoction for the treatment of cough [5]. Black cherry bark has been used by the Iroquois, Ojibwa, Malecite, and Delaware indigenous people from the North American boreal forest regions of Canada for treating diabetes-related symptoms [7].

Recently, our research group carried out chemical and pharmacological studies of the leaves, fruits and seeds of *P. serotine*. A bio-directed phytochemical study of the methanolic extract of the leaves led to the isolation of hyperoside and ursolic acid as the main vasodilator compounds. It was also found that benzyl alcohol, benzaldehyde, cinnamyl alcohol, and cinnamaldehyde were the major constituents of the essential oil obtained from the leaves, these compounds promoted vascular smooth muscle relaxation [8]. Regarding *P. serotina* fruits, we found that they have a high content of phenolic compounds such as chlorogenic acid, gallic acid, caffeic acid, catechin, epicatechin, quercetin, and kaempferol glycosides, which are directly related to the high antioxidant capacity and significant vasodilatory effect of the aqueous extract of the fruits [9]. Additionally, physicochemical and chemical characterization of black cherry seed oil demonstrated that it is mainly composed of polyunsaturated fatty acids, including oleic, linoleic, and α-eleostearic acids [10]. These results support the potential therapeutic significance of *P. serotina* seed oil, since it has been extensively demonstrated that consumption of unsaturated fatty acids reduce plasma lipids and reduce atherogenesis by decreasing inflammation of macrophages and vascular endothelial cells [11,12]. Moreover, it has been reported that α-eleostearic acid is effective in suppressing growth in cancer cells, and it has been proposed as a chemotherapeutic agent against breast cancer [13].

Although black cherry seeds contain cyanogenic glycosides [14], there are no reports of human intoxication related to their intake, since they are consumed toasted, which indicates that the toasting process decreases the content of these compounds [15]. Heat treatment also provokes emission of volatile compounds contained in the seeds, which undoubtedly contribute to their pleasant and characteristic flavor. These volatile components might also possess pharmacological activity [16].

Currently, the nutritional value of black cherry seeds and their volatile components have not been investigated. Therefore, in the present study the proximate composition, vitamin and mineral content of these seeds were determined in order to assess their nutritional value. In addition, the volatile compounds present in the seeds were analyzed by gas chromatography coupled to mass spectrometry.

## 2. Results and Discussion

### 2.1. Proximate Composition

Proximate compositions of raw and toasted black cherry seeds, *Prunus dulcis* (almonds) and *Arachis hypogaea* (peanuts) are shown in Table 1. There were no significant differences between protein, ash, crude fiber or carbohydrate contents of the raw and roasted seeds, suggesting that the toasting process does not affect the proximate composition. The mean values of protein content were 37.95% ± 0.16% and 36.55% ± 0.22% for raw and toasted seeds, respectively. These values were significantly higher than those of almonds (19.91%), and peanuts (22.82%), whose protein content is consistent with earlier reports [17,18]. *P. serotine* seeds possess a protein content which is comparable to that of seeds of other *Prunus* species, including apricots (*P. armeniaca* L., 37.4%), sweet cherries (*P. avium* L. 31.7%), sour cherries (*P. cerasus* L., 31.7%), nectarines (*P*. *persica ar. nectarina* (Aiton) Maxim, 38.7%), peaches (*P.* *persica* (L.) Batsch var. *persica*, 33.4%), and plums (*P. domestica* L., 35.9%) [19].

**Table 1:** Proximate composition of raw and toasted black cherry seeds, almond, and peanut.

Component (% Dry Basis)	Raw Black Cherry Seeds	Toasted Black Cherry Seeds	Almonds	Peanuts
**Moisture**	8.92 ± 0.42 ^a^	10.75 ± 0.35 ^a^	6.08 ± 0.4 ^b^	5.45 ± 0.38 ^c^
**Fat**	40.37 ± 0.73 ^a^	39.97 ± 0.20 ^a^	49.64 ± 0.42 ^b^	41.12 ± 1.51 ^a^
**Protein**	37.95 ± 0.16 ^a^	36.55 ± 0.22 ^a^	19.91 ± 0.01 ^b^	22.82 ± 0.01 ^c^
**Ash**	3.19 ± 0.18 ^a^	2.72 ± 0.21 ^a^	3.18 ± 0.21 ^a^	2.41 ± 0.19 ^a^
**Crude Fiber**	10.73 ± 1.49 ^a^	12.12 ± 4.06 ^a^	10.91 ± 1.45 ^a^	9.21 ± 1.59 ^a^
**Carbohydrates**	7.76 ± 2.24 ^a^	8.65 ± 4.28 ^a^	10.26 ± 1.98 ^b^	18.95 ± 2.68 ^c^

Notes: Data are given as mean ± standard deviation (*n* = 3 independent experiments performed in different samples). a, b and c: Values in the same row followed by the same superscript letter are not significantly different (*p* > 0.05).

Lipid content of *P. serotina* raw (40.37% ± 0.73%) and toasted seeds (39.97% ± 0.20%) was not significantly different from that of peanuts (41.12% ± 1.51%). However, it was significantly lower than almonds lipid content (49.64% ± 0.42%). It is well known that seed oils are mainly composed by mono- and polyunsaturated fatty acids, thus oleic (61%) and linoleic (29%) acids are the main fatty acids contained in almond; oleic acid (81%) is abundant in peanut oil [20], whereas, black cherry seed oil is rich in oleic (35%), linoleic (27%) and α-eleostearic (27%) acids [10].

These findings indicate that *P. serotina* edible seeds are a good source of unsatured fatty acids with potential health benefits [21]. The mean values of ash and crude fiber of raw and toasted *P. serotina* seeds, almonds and peanuts did not differ significantly. The crude fiber content was similar to that of other seeds and nuts that represent a complementary source of fiber, like hazelnut (9%–13%) or pumpkin seeds (12.1%) [22,23]. Adequate intake fiber is related to the obesity prevention and better glycemic control in patients with type 2 diabetes mellitus [24,25].

### 2.2. Minerals

The mineral composition (Ca, Fe, Mg, P, K, Zn and Na) of raw and toasted black cherry seeds, almonds and peanuts are presented in Table 2. The Mg content of raw and toasted black cherry seeds was unaffected by heat processing, but the Ca, Fe, P, K, Zn, and Na content diminished after the toasting process. Usually, nuts and seeds are consumed toasted and salted, and toasting might have an important influence on their nutrimental quality. Chitra *et al.* [26] and Gamel *et al.* [27] have pointed out that Ca and Fe content significantly reduced after toasting or autoclaving seeds of soybean and pigeon peas.

**Table 2:** Mineral composition of raw and toasted black cherry seeds, almond and peanut.

Mineral (Dry Basis) mg/100 g	Raw Black Cherry Seeds	Toasted Black Cherry Seeds	Almonds	Peanuts
**Ca**	192.30 ± 0.58 ^a^	127.11 ± 17.51 ^b^	305.43 ± 1.88 ^c^	91.43 ± 0.05 ^d^
**Fe**	9.49 ± 0.3 ^a^	1.21 ± 0.003 ^b^	6.08 ± 0.01 ^c^	8.31 ± 0.02 ^d^
**Mg**	249.15 ± 0.34 ^a^	216.68 ± 18.75 ^a^	282.09 ± 0.32 ^b^	172.75 ± 0.77 ^c^
**P**	439.0 ± 0.16 ^a^	323.40 ± 0.14 ^b^	387.03 ± 0.7 ^c^	347.41 ± 0.07 ^d^
**K**	873.22 ± 12.64 ^a^	454.82 ± 0.41 ^b^	656.25 ± 23.80 ^c^	571.57 ± 10.03 ^d^
**Zn**	3.40 ± 0.10 ^a^	2.96 ± 0.24 ^b^	4.48 ± 0.17 ^c^	3.92 ± 0.10 ^d^
**Na**	82.98 ± 0.90 ^a^	23.59 ± 0.8 ^b^	76.57 ± 0.38 ^a^	62.99 ± 0.65 ^c^

Notes: Data are given as mean ± standard deviation (*n* = 3 independent experiments performed in different samples). a, b, c and d: Values in the same row followed by the same superscript letter are not significantly different (*p* > 0.05).

Raw black cherry seeds possess a higher content of Ca, Fe, Mg, K and Na than that found in peanuts. On the other hand, Fe, K, and Na content is greater than that of almonds. Interestingly, the K content in black cherry seeds (873.22 ± 12.64 mg/100 g) is significantly higher than that of almonds and peanuts, suggesting that *P. serotina* seeds represent a good complementary source of this mineral.

### 2.3. Vitamins

Vitamin analysis showed that raw and toasted *P. serotina* seeds do not contain vitamins A and C. Vitamin E or α-tocopherol was detected at a concentration of 3.916 mg/100 g in raw seeds, while in toasted seed it was absent. This lost may be associated with vitamin degradation due to temperature exposure [27]. Our results are in agreement with previous studies carried out on other seeds and nuts, such as pumpkin seeds, walnuts and peanuts, which do not contain detectable values of vitamins A and C [28,29]. Vitamin E content of black cherry seeds is similar to that of pine nuts (4.1 mg/100 g) and peanuts (6.1 mg/100 g), however it is lower than that of almonds and hazelnuts (24.2 mg/ 100 g and 31.4 mg/ 100 g, respectively) [28].

### 2.4. Protein Nutritional Quality

Since black cherry seeds contain higher levels of protein (37.95% ± 0.16%) than other seeds, their protein nutritional quality was assessed. Amino acid composition data are presented in Table 3. It is important to mention that in this experiment tryptophan was not determined.

**Table 3:** Protein amino acids composition of raw and toasted black cherry seeds.

Amino Acid	Raw Seeds mg/g Protein	Toasted Seeds mg/g Protein
Asp	112.29	116.97
Glu	256.84	273.73
Ser	32.84	42.11
His	21.60	21.29
Gly	37.43	38.82
Thr	52.85	59.16
Arg	84.24	87.42
Ala	41.47	44.06
Tyr	48.75	60.99
Met	8.93	9.83
Val	45.48	45.62
Phe	48.64	52.00
Ile	39.17	40.33
Leu	75.10	82.11
Lys	8.85	11.17

Another protein quality measure is digestibility. Differences among the *in vitro* protein digestibility values of raw and toasted black cherry seeds were not significant (88.12% ± 0.72% and 89.40% ± 1.32%, respectively). These values were similar to that of almonds (90.15% ± 0.85%), however they were lower than those of peanuts (94.06% ± 0.78%) and casein (98.51% ± 0.82%), which was used as control. It has been reported that *in vitro* protein digestibility values greater than 80% are related to an efficient amino acid bioavailability [27]. Therefore, these results suggest that black cherry seed proteins are highly bioavailable.

Considering these findings, the protein digestibility-corrected amino acid score (PDCAAS) was determined. This method is accepted as the most recognized approach for assessing the protein quality of foods [30]. Every essential amino acid receives a score, but the final PDCAAS value corresponds to that of the limiting amino acid. The highest PDCAAS value for a given protein is 1.0, which indicates that a protein provides adequate amounts of all the essential amino acids [31]. Table 4 shows that lysine is the limiting amino acid in *P. serotina* seeds, therefore, PDCAAS values for raw and toasted black cherry seeds protein are 0.13 and 0.18, respectively. These values are lower than those reported for almonds (0.23) [17] and peanuts (0.69) [32]. These results suggest that black cherry seeds could be a complementary source of proteins.

**Table 4:** Black cherry seeds amino acid score (AAS).

AA	FAO Reference Pattern	AAS *	AAS *
		Raw Seeds	Toasted Seeds
His	19	1.37	1.12
Thr	34	1.55	1.74
Met	25	0.36	0.39
Val	35	1.30	1.33
Phe	63	0.77	0.82
Ile	28	1.40	1.44
Leu	66	1.38	1.24
Lys **	58	0.15	0.19

Notes: * Amino acid score (AAS) = AA content of test protein/reference AA pattern, where reference AA pattern is the amino acid requirement for a preschool child (2–5 years) [30]. ** Limiting amino acid.

### 2.5. Volatile Compounds

Volatile compounds of raw and toasted black cherry seeds were extracted by head space solid phase micro extraction and analyzed by gas chromatography-mass spectrometry, using DB-5 and Wax capillary columns in order to detect a wide range of polar and non-polar compounds. A total of 59 and 99 volatile compounds were identified in the raw and toasted *P. serotina* seeds, respectively (Table 5). The identified volatile compounds comprise aldehydes, alcohols, ketones, carboxylic acids, esters, hydrocarbons, and pyrazines.

The predominant aldehyde identified in raw and toasted seeds was benzaldehyde. This compound has a characteristic pleasant almond-taste and aroma [33] and has been identified in bitter and sweet almonds [34], hazelnuts [33], and seeds of other species of the genus *Prunus* [35,36]. Benzaldehyde is produced by enzymatic degradation of the cyanogenic glycosides amygdalin and prunasin [35,37,38]. Considering on one hand that benzaldehyde content in toasted seeds was higher than that in raw seeds, and on the other, that bitterness attributed to the presence of amygdalin [39,40] diminished in toasted seeds, it is very likely that the toasting process may induce benzaldehyde formation from cyanogenic glycosides. Benzaldehyde might also be generated from phenylalanine during heating processes [41,42] as was demonstrated by Alasalvar *et al.* [33] in hazelnuts, pumpkin seeds [43], and peanuts [44]. It is worth noting that we have previously identified benzaldehyde as one of the major components in black cherry leaves essential oil, we also proved that this compound induces a significant concentration-dependent vasodilator effect [8]. Therefore, the presence of this compound along with oleic, linoleic, and α-eleostearic fatty acids indicate that black cherry seeds consumption might have beneficial effects on the cardiovascular system.

**Table 5:** Volatile compounds on raw and toasted black cherry seeds.

RI Calculated ^a^		RI Literature ^b^		Compound	Raw ^c^	Toasted ^c^	ID ^e^
DB5	WAX	DB5	WAX		(ppm ^d^)	(ppm ^d^)	
**Aldehydes**							
573	805		801	Propanal, 2-methyl-		0.604	MS, RI
638		640		2-Butenal		0.180	MS, RI
642	908	642		Butanal, 3-methyl-		1.095	MS, RI
652	910	653		Butanal, 2-methyl-		1.419	MS, RI
695	965	695	970	Pentanal		2.065	MS, RI
740		740		2-Butenal, 2-methyl-		0.500	MS, RI
	1074		1078	Hexanal		0.096	MS, RI
834	1440	835	1443	Furfural		2.609	MS, RI
902		905		Heptanal		0.084	MS, RI
908		909		Propanal, 3-(methylthio)-		0.041	MS, RI
	1199		1196	2-Hexenal, (*E*)-		0.014	MS, RI
964		963		2-Furancarboxaldehyde, 5-methyl-		0.112	MS, RI
967	1508	969	1508	Benzaldehyde	1.371	7.597	MS, RI
1053		1053		Benzeneacetaldehyde		0.411	MS, RI
1106		1108		Nonanal	0.056	0.201	MS, RI
	1443		1455	3-Furaldehyde	0.090		MS, RI
1221	1697	1221	1697	2,4-Nonadienal, (*E*,*E*)-		0.091	MS, RI
1279	1930	1279	1933	Benzeneacetaldehyde, alpha.-ethylidene-		0.045	MS, RI
**Ketones**							
707	1303	708	1301	2-Butanone, 3-hydroxy-	0.329	0.361	MS, RI
986		986		3-Octanone	0.055		MS, RI
1027		1043		1,2-Cyclopentanedione, 3-methyl-		0.221	MS
1067	1958	1067	1957	Ethanone, 1-(1*H*-pyrrol-2-yl)-		0.233	MS,RI
1071		1071.6		Acetophenone		0.051	MS,RI
1114		1117		1,7-Octadien-3-one, 2-methyl-6-methylene-	0.037		MS,RI
	1448			Cyclohexanone, 5-methyl-2-(1-methylethyl)-, (2*S*-*trans*)-	0.037		MS
	1486		1489	Ethanone, 1-(2-furanyl)-		0.119	MS, RI
	1487			1-(3*H*-Imidazol-4-yl)-ethanone		0.226	MS
1151		1151		4*H*-Pyran-4-one, 2,3-dihydro-3,5-dihydroxy-6-methyl-		0.107	MS, RI
	1569		1573	2-Cyclopentene-1,4-dione		1.536	MS, RI
	1588		1587	Ethanone, 1-(2-pyridinyl)-		0.526	MS, RI
	1814			1,2-Cyclopentanedione, 3-methyl-		0.134	MS
	2012			2(3*H*)-Furanone, dihydro-3-hydroxy-4,4-dimethyl-		0.101	MS
**Carboxylic acids**							
613	1415	610	1415	Acetic acid	2.163	5.939	MS, RI
	1608			Benzoic acid, hydrazide	0.040		MS
	1616			Butanoic acid, 4-hydroxy-	0.184	0.656	MS
	1829		1827	Hexanoic acid	0.282	0.089	MS, RI
	2028		2030	Octanoic Acid	0.110	0.209	MS, RI
**Esters**							
719	972	720	971	Butanoic acid, methyl ester	0.019		MS, RI
774	1007	776	1007	Acetic acid, 2-methylpropyl ester	0.191	0.096	MS, RI
826	1076	825	1075	Pentanoic acid, methyl ester	0.166		MS, RI
878	1115	877	1115	1-Butanol, 3-methyl-, acetate	0.282	0.404	MS, RI
880		880		1-Butanol, 2-methyl-, acetate		0.260	MS, RI
912	1616	912	1617	Butyrolactone	0.297		MS, RI
925		926		Hexanoic acid, methyl ester	0.120		MS, RI
	1320		1317	Heptanoic acid, ethyl ester	0.061		MS, RI
1045				Pantolactone		0.192	MS
1099	1607	1100	1605	Benzoic acid, methyl ester	0.162		MS,RI
1167		1165		Acetic acid, phenylmethyl ester		0.096	MS,RI
**Alcohols**							
615	1085	618	1086	1-Propanol, 2-methyl-	0.389	0.229	MS, RI
	918		920	Isopropyl Alcohol	0.080		MS, RI
734	1189	730	1190	1-Butanol, 3-methyl-	0.173		MS, RI
738	1189			1-Butanol, 2-methyl-, (+/−)-	0.381		MS
767	1231	766	1232	1-Pentanol	0.595	0.536	MS, RI
797	1584	800	1583	2,3-Butanediol [*R*-(R*,R*)]-	17.039	17.448	MS, RI
	1088			1,2-Cyclopentanediol, *trans*-		0.030	MS
	1136		1136	1-Butanol	0.050	0.062	MS, RI
858	1635	858	1638	2-Furanmethanol		0.747	MS, RI
955	1700	953		2-Furanmethanol, 5-methyl-		0.096	MS, RI
	1334		1335	1-Hexanol	0.201		MS, RI
1039	1866	1039	1866	Benzyl alcohol	0.332	1.019	MS, RI
1051				3-Octen-1-ol	0.069		MS
	1379		1379	Ethanol, 2-butoxy-	0.063		MS, RI
	1580			Cyclohexanol, 5-methyl-2-(1-methylethyl)-, (1α, 2β, 5α)-		0.743	MS
	1885			3,6,9,12-Tetraoxatetradecan-1-ol	0.008		MS
	1903		1905	Phenylethyl Alcohol	0.065	0.166	MS, RI
1684				2,6-Bis(1,1-dimethylethyl)-4-(1-oxopropyl)phenol	0.010	0.014	MS
**Hydrocarbons**							
647	923	648	924	Benzene	0.043	0.086	MS, RI
765	1029	765	1029	Toluene	0.052	0.110	MS, RI
	1094			Undecane	0.030		MS
	1126		1123	Benzene, 1,3-dimethyl-	0.050		MS, RI
862		863		Ethylbenzene	0.022		MS, RI
872				Cyclopropane, propyl-		0.324	MS
	1166		1164	*p*-Xylene	0.010		MS, RI
893	1237	894	1236	Styrene	0.108	0.084	MS, RI
899				Nonane	0.020	0.074	MS
931				2,4-Octadiene		0.057	MS
	1238		1264	1,3,5,7-Cyclooctatetraene		0.300	MS
996		996		Benzene, 1,3,5-trimethyl-	0.040		MS, RI
999	997			Decane	0.096	0.089	MS
1033	1079	1030		D-Limonene	0.025	0.048	MS, RI
	1412			Benzene, 2-ethyl-1,4-dimethyl-	0.011		MS
	1413			Benzene, 1-ethyl-2,3-dimethyl-	0.027		MS
1125		1120		Benzene, 1,2,3,4-tetramethyl-	0.040		MS, RI
1303		1300		Tridecane	0.015	0.027	MS, RI
1399		1400		Tetradecane	0.015	0.040	MS, RI
	2056			1,4,7,10,13,16-Hexaoxacyclooctadecane	0.029		MS
**Pyrazines**							
826	1247	827	1247	Pyrazine, methyl-		3.745	MS, RI
896	1487		1491	2,3,5-Trimethyl-6-ethylpyrazine		0.221	MS, RI
915	1306	915	1308	Pyrazine, 2,5-dimethyl-		7.344	MS, RI
	1312		1314	Pyrazine, 2,6-dimethyl-		1.297	MS, RI
998	1365	998	1367	Pyrazine, 2-ethyl-6-methyl-		0.701	MS, RI
1002	1384	1005	1381	Pyrazine, trimethyl-		1.685	MS, RI
	1330		1326	Pyrazine, 2,3-dimethyl-		0.287	MS, RI
	1371		1367	Pyrazine, 2-ethyl-5-methyl-		1.494	MS, RI
1078	1425	1078	1430	Pyrazine, 3-ethyl-2,5-dimethyl-		1.907	MS, RI
1085		1085		Pyrazine, 2-ethyl-3,5-dimethyl-		0.143	MS, RI
1086	1458	1088	1457	Pyrazine, tetramethyl-	0.106	0.597	MS, RI
	1411		1415	Pyrazine, 2,6-diethyl-		0.066	MS, RI
1094		1091		Pyrazine, 2,5-diethyl-		0.106	MS, RI
	1472		1485	Pyrazine, 2-ethenyl-6-methyl-		0.105	MS, RI
1147	1609		1605	5*H*-5-Methyl-6,7-dihydrocyclopentapyrazine		0.500	MS, RI
1153	1475	1156	1478	Pyrazine, 2,3-diethyl-5-methyl-		0.130	MS, RI
1157		1156		Pyrazine, 3,5-diethyl-2-methyl-		0.245	MS, RI
1161		1357		2,5-Dimethyl-3-n-pentylpyrazine		0.021	MS
	1683		1679	1-(6-Methyl-2-pyrazinyl)-1-ethanone		0.146	MS, RI
1318		1254		Pyrazine, 2-butyl-3,5-dimethyl-		0.019	MS
	1705		1740	Pyrazinamide		0.208	MS
**Pyrroles and Furans**							
811	1165	811	1168	1*H*-Pyrrole, 1-ethyl-		0.144	MS, RI
847		841		1*H*-Pyrrole, 3-methyl-		0.082	MS, RI
948				1*H*-Pyrrole, 1-butyl-		0.021	MS
891	1122	892.1	1123	2-*n*-Butylfuran		0.707	MS, RI
991	1211	991	1213	Furan, 2-pentyl-	0.077	0.236	MS, RI
	1037			2,3-Dihydrofuran		0.008	MS
1016	2004	1016	2006	1*H*-Pyrrole-2-carboxaldehyde		0.114	MS, RI
1076	2020	1076	2020	2-Pyrrolidinone		0.477	MS, RI
	1675		1678	2-Pyrrolidinone, 1-methyl-		0.057	MS, RI
**Miscellaneous**							
	632		643	Methanethiol		0.046	MS
559	701			Dimethyl sulfide	0.052	0.071	MS
567		531		Methylene chloride	0.844	0.474	MS
612	1009	615	1010	Trichloromethane	0.059	0.028	MS, RI
724				Propanamide*, N,N*-dimethyl-	0.025		MS
850		850		Oxazole, trimethyl-		0.034	MS, RI
1020		1020		Benzene, 1,4-dichloro-	0.450	0.253	MS, RI
	1419			Benzene, 1,2-dichloro-		0.450	MS
	1710			6-Aminoindoline		0.136	MS
	1923		1912	Benzonitrile, 2-methyl-		0.060	MS
1537				*N*-(2-Benzoyl-4-nitrophenyl)-4-*tert*-butylbenzamide		0.041	MS
	2051			Octaethylene glycol	0.007		MS

Notes: ^a^ Calculated Kovats retention index on DB-5 or Wax column using a n-alkanes series; ^b^ Reported Kovats retention index on library NIST; ^c^ Average of 3 replicates. Coefficient of variation < 9.2%; ^d^ Semi-quantitative values. ^e^ MS = tentative identification by comparison to mass spectra in NIST library, RI = identification by comparison of calculated Kovats retention index to that reported in literature.

The most abundant alcohol identified in *P. serotina* seeds was 2,3-butanediol, which was also the major volatile compound in raw (17.03 ppm) and toasted seeds (17.44 ppm). This alcohol is the major volatile component of sweet kernels and has been associated to their characteristic taste [34]. 2,3-butanediol is also found in cheeses [45] and honey [46]. Recently, it has been reported that this metabolite is abundant in roasted *Trichosanthes kirilowii* seeds, however it was absent in the raw seeds, which suggests that 2,3-butanediol was generated during the toasting process [47]. In contrast, our results indicated that 2,3-butanediol levels in black cherry seeds were not significantly different before and after toasting. Benzyl alcohol, predominantly found in toasted seeds, imparts a sharp, burning taste and a faint aroma in toasted almonds [48]. This compound was previously detected as one of the major volatiles of black cherry leaves and also displays a vasodilator effect [8]. Regarding 1-pentanol, detected in both raw and toasted seeds, it has been proposed that it derives from the oxidation of linoleic acid, and its concentration increases with storage time in almonds [49].

Acetic acid was the main volatile carboxylic acid found in raw and toasted *P serotina* seeds. This carboxylic acid can be regarded as a result of long-chain fatty acids degradation and it is often related to a strong sour odor [50]. Esters were detected in lesser quantities, some of them, such as methyl esters of short chain fatty acids, were found in raw seeds, but not in toasted seeds. Hydrocarbons and ketones were minor compounds in both raw and toasted seeds.

Pyrazines and pyrroles were other major volatiles identified exclusively in toasted black cherry seeds. These compounds are produced during the toasting process from free amino acids and monosaccharides by the Maillard reaction through Strecker degradation [33], particularly, ethyl-methyl‑, ethyldimethyl-, and diethylmethylpyrazines, are formed in the reaction of glucose and fructose with alanine and glycine [51]. The most abundant pyrazine in black cherry toasted seeds was 2,5-dimethylpyrazine, followed by methylpyrazine, 3-ethyl-2,5-dimethylpyrazine and trimethylpyrazine. The first one has been previously reported as a major pyrazine in roasted hazelnuts [33] and toasted almonds [48]. Pyrazines contribute to the characteristic toasted aroma in chesnuts [52], and peanuts [53], almonds [54], and pyrroles significantly are responsible for the characteristic toasted aroma of different thermal treated foods [44].

## 3. Experimental Section

Reagents and standards were purchased from Sigma-Aldrich (St. Louis, MO, USA), unless otherwise noted. Solvents were purchased from Baker-Mallincrodt (JT Baker, Mallinckrodt Baker Inc., Phillipsburg, NJ, USA).

### 3.1. Samples

Ripe black cherry fruits were cultivated in Huejotzingo, Puebla (México) and harvested in May of 2011. Subsequently, the seeds were removed from the pulp with plastic knives, washed and allowed to dry at 25 °C during 48 h. Finally, the seeds were stored at −70 °C until analysis.

All the analyses were carried out on raw and toasted seeds. Toasting was performed in a *comal* (traditional Mexican iron griddle) during 20 min at 125 °C. Then, the seeds were rotated frequently to avoid overheating and to obtain homogenous toasting. Raw and toasted seeds were cracked and opened with pliers to get the kernel.

Taking into account that black cherry toasted seeds are consumed by Mexican people as snacks, almonds (*Prunus dulcis*) and peanuts (*Arachis hypogaea*) samples were analyzed for comparative purposes. Both seeds were purchased in a local market in the city of Queretaro, Queretaro (México).

### 3.2. Chemical Proximate Analysis

Chemical proximate analysis was carried out on raw and toasted black cherry seeds, almonds and peanuts by using AOAC methods (AOAC, 2000). Moisture, protein, fat, ash, crude fiber and carbohydrates contents were determined by methods 950.46, 928.08, 960.39, 920.153 and 985.29, respectively. Total carbohydrate content (on dry weight basis) was calculated by difference [100 − (protein + lipids + ash + crude fiber)].

### 3.3. Determination of Vitamins A, C, and E

Vitamins and carotenes were determined by using AOAC methods (2000) for vitamin A (960.46), vitamin C (967.22), and vitamin E (992.03).

### 3.4. Mineral Content

Sodium, potassium, calcium, magnesium and iron content of samples was determined by method 985.35 described in Association of Official Analytical Chemists (AOAC, 2000). Phosphorous was determined according to method 965.17 (AOAC, 2000).

### 3.5. In Vitro Protein Digestibility

The *in vitro* protein digestibility of dry samples was estimated by using the methodology of Hsu *et al.* [55] and applying the equation *Y* = 234.84 − 22.56*X*, where *Y* is the *in vitro* protein digestibility (%) and *X* is the pH of the protein sample suspension, after proteolysis with a multienzyme system consisting of porcine pancreatic trypsin type IX, bovine pancreatic chymotrypsin type II, and porcine intestinal peptidase grade III. The average value of three replicates is reported.

### 3.6. Amino Acid Analysis

Proteins were hydrolyzed in hydrochloric acid (JT Baker) and amino acids were analyzed using a HPLC autoanalyzer (Waters 2487, Millipore, MA, USA), according to Bidlingmeyer *et al.* [56].

### 3.7. Nutritional Quality of Proteins

For predicting dietary protein quality, the *in vitro* protein digestibility and amino acid composition were used to calculate PDCAAS (protein digestibility corrected amino acid score) according to the FAO/WHO [30] prescribed formula:

PDCAAS = Amino acid score (of the most limiting AA) × Digestibility
(1)

### 3.8. Volatile Compounds Analysis

Extraction conditions were based on previously published work of Krist *et al.* [52] and Agila *et al.* [54] with some modifications. Fresh seeds (15 g) were blended in an electric blender (model O-20, Osterizer^TM^, Boca Raton, FL, USA) for 15 s. The ground seeds (1 g) were transferred into a 20 mL vial, along with 7 µg of a menthol solution (0.01% w/w) as an internal standard and sealed with a Nickel-aluminum crimp cap provided with a needle-pierceable polytetrafluroethylene/silicone septum. Vials were pre-equilibrated for 60 min at 50 °C. Solid-phase microextraction (SPME) was performed with a 75 mm divinylbenzene/carboxen/polydimethylsiloxane (DVB/CAR/PDMS) fiber (Supelco Co, Bellefonte, PA, USA). Extractions and injections were performed using a MPS2 autosampler (Gerstel, Linthicum-Baltimore City, MD, USA) fitted with a vial heater. The fiber was exposed for 10 min in the head space of the vial for analytes adsorption. Subsequently, the fiber was removed from the vial and placed into the chromatograph injection port for desorption.

Gas chromatography separation and quantification was carried out using an Agilent GC 7890A series intrumenbt (Agilent Technologies, Inc., Santa Clara, CA, USA) equipped with a flame ionization detector (GC-FID). Injection port and detector temperature was 230 °C. The injector was operated in the splitless mode. The capillary columns used were an HP-5 60 m × 0.32 mm i.d., 0.32 µm film thickness; Agilent Technologies, Inc.) and a DB-Wax (60 m × 0.32 mm i.d., 0.32 µm film thickness; Agilent Technologies, Inc.). Oven temperature was programmed at initial temperature of 40 °C for 5 min, then raised at 5 °C/min to 230 °C and hold for 15 min. Helium was used as the carrier gas at a constant flow rate of 1 mL/min. A semi-quantitative evaluation was achieved by comparing individual peak area from GC-FID response to that of the internal standard. Each tabulated value corresponds to the average of 3 extraction replicates.

Gas Chromatography Mass Spectrometry (GC/MS) analysis was carried out using an Agilent GC 7890A series equipped with an Agilent 5975C Mass Spectrometer in electron impact mode (EI) and a quadrupole analyzer. The temperatures of ion source and quadrupole were 230 and 250 °C respectively. Transfer line was set at 280 °C. Full scan mode was used in a range of 33–300 uma, at a scan rate of 5.2/s, with an ionization voltage of 70 eV. The same chromatographic conditions as for GC-FID were used. MSD ChemStation E.01.00.237 software (Agilent Technologies, Inc.) was employed for data analysis.

Identification of volatile compounds was performed by comparing their mass spectra with those in the NIST Mass Spectral Library (National Institute of Standards and Technology, Gaithersburg, MD, USA). Retention indices of all the volatile compounds were determined by the modified Kovats method reported by Van den Dool and Kratz [57]. MS identification was confirmed by comparing Kovats retention indices (RI) to RI reported in the literature [58].

### 3.9. Statistical Analysis

Results of the experiments are expressed as the mean ± standard deviation (SD) from *n* = 3 experiments. The data were analyzed by a one-way ANOVA and the Tukey test. Differences between the means were considered to be significant when *p* ˂ 0.05. Statistical treatment of data was performed with the program Prism 4.0 (GraphPad Software, San Diego, CA, USA).

## 4. Conclusions

This study demonstrates that black cherry seeds have a significant content of minerals, particularly potassium, lipids and proteins. Their PDCAAS values suggest that they might be considered as a complementary source of protein. Additionally, the gas chromatography/mass spectrometry analysis allowed identification of 59 and 99 volatile compounds in the raw and toasted seeds, respectively. The major volatile components identified in raw and toasted *P. serotina* seeds were 2,3-butanediol and benzaldehyde. The results derived from this study indicate that these seeds have nutraceutical properties attributed to their high protein and potassium content and the presence of bioactive compounds such as benzaldehyde.
